# Pseudo-subarachnoid Hemorrhage

**DOI:** 10.5334/jbsr.1509

**Published:** 2018-03-01

**Authors:** Bruno Coulier

**Affiliations:** 1 Clinique Saint-Luc, Bouge, BE

**Keywords:** Pseudo-subarachnoid hemorrhage, Brain computed tomography (CT), cerebral edema

## Case

**Figure 1:**
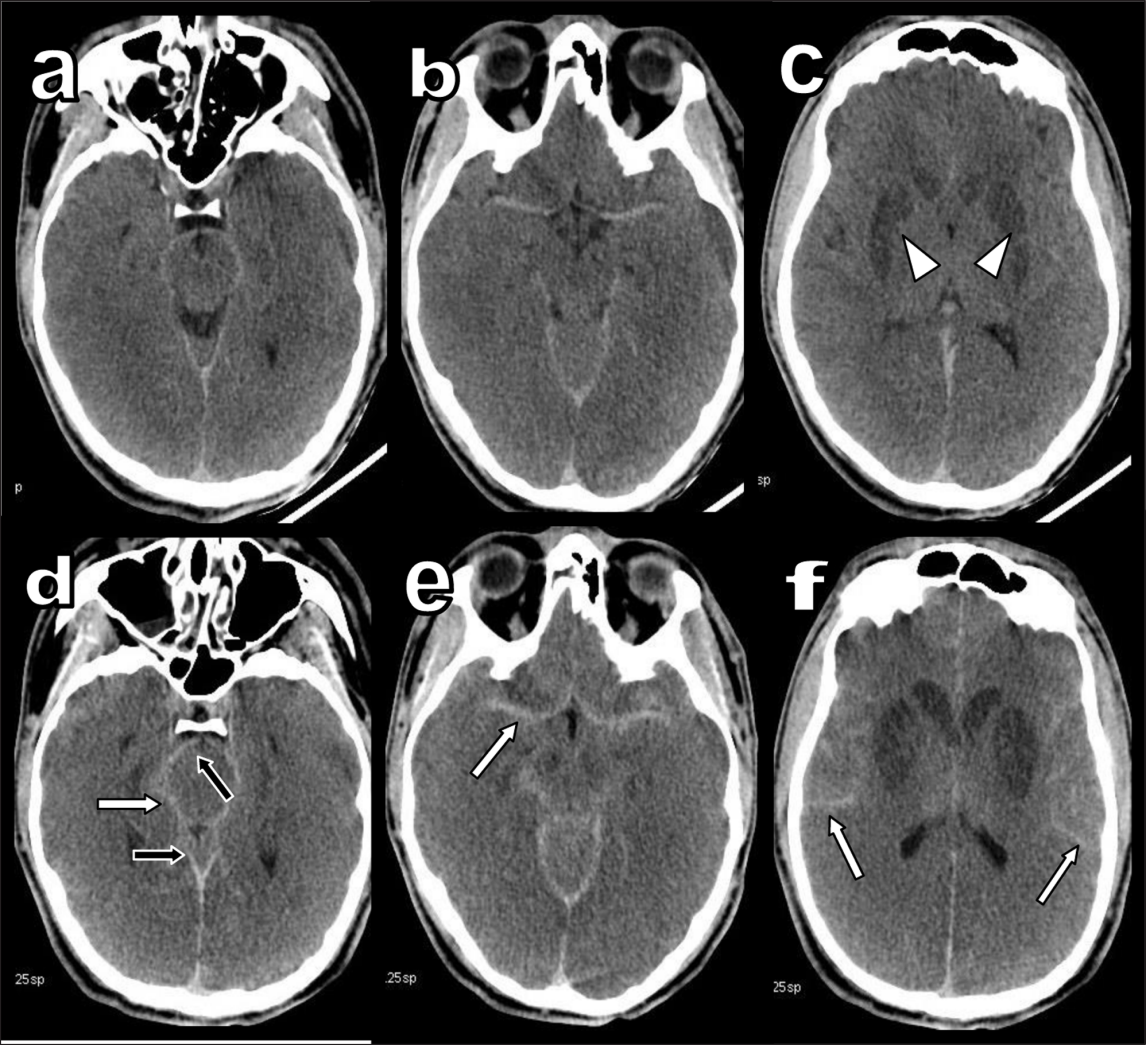
Comparison of unenhanced Brain CT (UBCT) obtained 5 hours after admission **(a to c)** and 30 hours after admission **(d to f)**.

**Figure 2:**
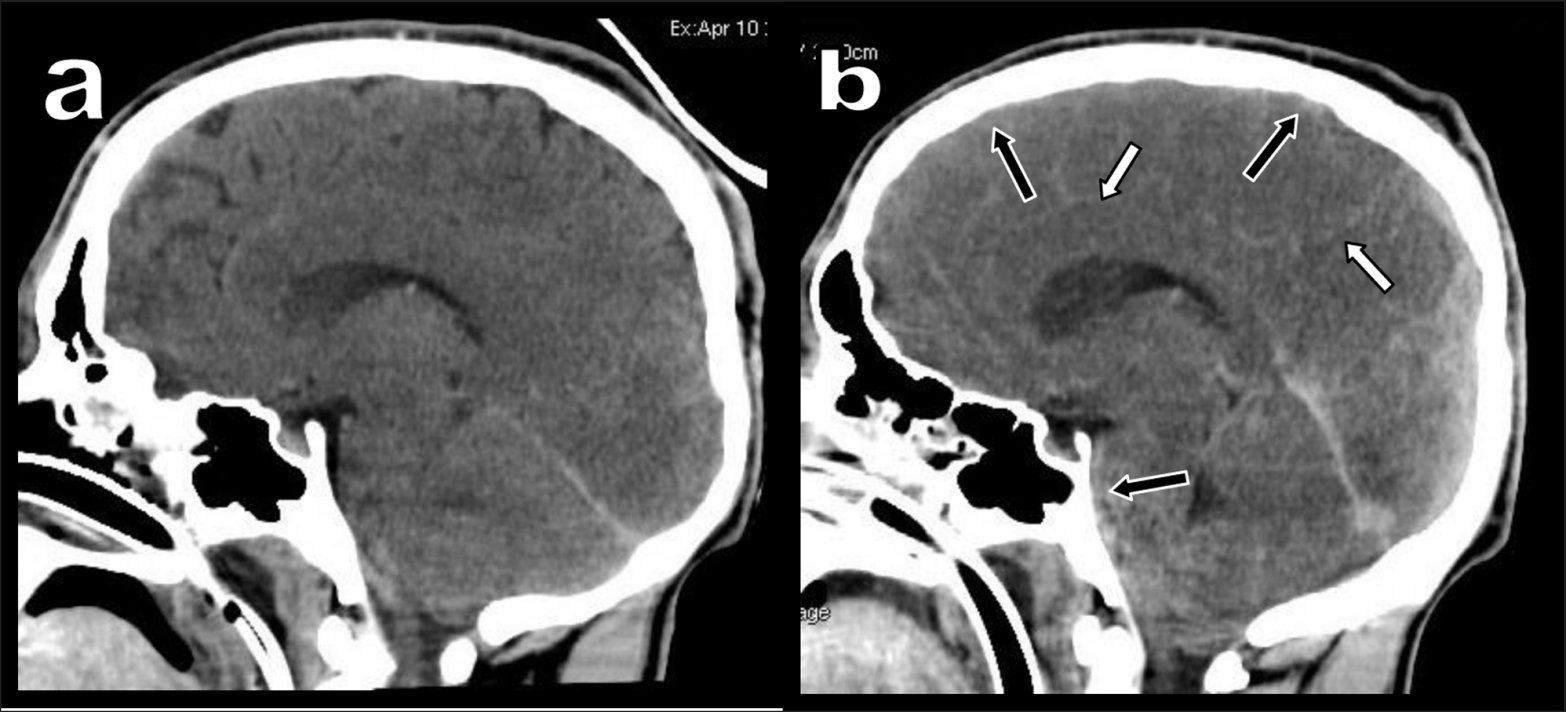
Comparison of median sagital views of unenhanced Brain CT at 5 hours **(a)** and 30 hours **(b)**.

A 19-year-old man was admitted in the intensive unit after a suicide attempt by hanging. He had been found in cardiac arrest of imprecise duration. Cardiac massage was performed for 40 minutes during transfer. The patient was in a deep coma with Glasgow score at 3/15. Unenhanced Brain Computed Tomography (CT) performed after five hours (Figures 1a, b, c and 2a) demonstrated bilateral hypodensity of the basal ganglia (white arrowheads). Hyperdensity of the cerebral arteries seemed to be related to the underlying brain hypodensity, with decrease in gray-white differentiation due to edema. Unenhanced CT after 30 hours (Figures 1e, f, g and 2b) showed progression of brain edema with collapse of the sylvian fissures, basal cisterns and cortical sulci (black arrows). Diffuse hyperdensity of all collapsed sub-arachnoid spaces had become prominent (white arrows) evocating pseudo-subarachnoid hemorrhage (PSAH). Multiple evoked potential confirmed brain dead after 48 hours.

## Comment

PSAH is an uncommon phenomenon in which hyper attenuation of cerebral sulci, fissures and cisterns mimicking true subarachnoid hemorraghe is found on unenhanced brain CT of patients presenting mostly with severe brain edema but in which no blood is found after lumbar puncture or at autopsy.

PSAH has also been reported in patients with leptomeningeal diseases, intracranial hypotension, cerebellar infarctions, bilateral subdural haematomas or in cases related to iatrogenic causes (intrathecal administration of contrast material or leakage of high-dose of intravenous contrast medium within the sub-arachnoid spaces).

Causes for brain edema rarely include toxic or metabolic encephalopathies but mostly, massive hypoxic or anoxic encephalopathy. PSAH has recently been observed in as much of 20% of patients resuscitated from non-traumatic cardiopulmonary arrest [1]. PSAH found within 6 hours after return of spontaneous circulation in these survivors appears very specific of a poor neurologic outcome and plays a precious early pronostic role.

Several causes may explain PSAH. Severe brain edema compresses the dural sinuses and compromises the venous drainage with secondary engorgement of the superficial veins. Their spontaneous hyperdensity is synergistically enlightened by the increased hypodensity of the adjacent brain, with loss of gray-white matter differentiation. The associated narrowing and/or complete collapse of the hypodense cerebrospinal fluid spaces is an unquestionable additional amplifier. The 30–42 UH mean density found in PSAH remains significantly lower than the 60–70 UH density of fresh blood in true subarachnoid hemorrhage. Furthermore, the absence of intraventricular blood is also typical of PSAH.

Strangulation and/or suicidal hanging and/or cardiac arrest are the main causes of cerebral hypoxia-anoxia in adults. The term “near hanging” refers to patients who survive a hanging injury long enough to reach hospital. Only few persons survive this episode and hypoxic encephalopathy is by far the most common cause of delayed hanging death.
